# Comparisons of three polyethyleneimine-derived nanoparticles as a gene therapy delivery system for renal cell carcinoma

**DOI:** 10.1186/1479-5876-9-46

**Published:** 2011-04-23

**Authors:** Zhizhong Xu, Guobo Shen, Xiangying Xia, Xinyu Zhao, Peng Zhang, Huanhuan Wu, Qingfa Guo, Zhiyong Qian, Yuquan Wei, Shufang Liang

**Affiliations:** 1 State Key Laboratory of Biotherapy and Cancer Center, West China Hospital, West China Medical School, Sichuan University, Chengdu, 610041, P. R. China; 2 Department of Urinary Surgery, West China Hospital, West China Medical School, Sichuan University, Chengdu 610041, China; 3 School of Chemical Engineering, Sichuan University, Chengdu, 610041, P. R. China

**Keywords:** Polyethyleneimine, nanoparticle, gene delivery, VHL, renal cell carcinoma

## Abstract

**Background:**

Polyethyleneimine (PEI), which can interact with negatively charged DNA through electrostatic interaction to form nanocomplexes, has been widely attempted to use as a gene delivery system. However, PEI has some defects that are not fit for keeping on gene expression. Therefore, some modifications against PEI properties have been done to improve their application value in gene delivery. In this study, three modified PEI derivatives, including poly(ε-caprolactone)-pluronic-poly(ε-caprolactone) grafted PEI (PCFC-g-PEI), folic acid-PCFC-isophorone diidocyanate-PEI (FA-PEAs) and heparin-PEI (HPEI), were evaluated in terms of their cytotoxicity and transfection efficiency *in vitro *and *in vivo *in order to ascertain their potential application in gene therapy.

**Methods:**

MTT assay and a marker GFP gene, encoding green fluorescent protein, were used to evaluate cell toxicity and transfection activity of the three modified PEI *in vitro*. Renal cell carcinoma (RCC) models were established in BALB/c nude mice inoculated with OS-RC-2 cells to detect the gene therapy effects using the three PEI-derived nanoparticles as gene delivery vehicles. The expression status of a target gene Von Hippel-Lindau (VHL) in treated tumor tissues was analyzed by semiquantitative RT-PCR and immunohistochemistry.

**Results:**

Each of three modified PEI-derived biomaterials had an increased transfection efficiency and a lower cytotoxicity compared with its precursor PEI with 25-kD or 2-kD molecule weight *in vitro*. And the mean tumor volume was obviously decreased 30% by using FA-PEAs to transfer VHL plasmids to treat mice RCC models. The VHL gene expression was greatly improved in the VHL-treated group. While there was no obvious tumor inhibition treated by PCFC-g-PEI:VHL and HPEI:VHL complexes.

**Conclusions:**

The three modified PEI-derived biomaterials, including PCFC-g-PEI, FA-PEAs and HPEI, had an increased transfection efficiency *in vitro *and obviously lower toxicities compared with their precursor PEI molecules. The FA-PEAs probably provide a potential gene delivery system to treat RCC even other cancers in future.

## Introduction

Renal cell carcinoma (RCC) is the third most common urological cancer with an incidence of approximately 5-10 per 100,000 and comprises 2-3% of all malignancies [1]. The majority (~80%) type is defined as clear cell RCC (CCRCC) which has bad prognosis and does not sensitive to radiotherapy and chemotherapy [2,3]. Therefore, a novel therapy strategy against CCRCC needs to be developed. In the past decade, gene therapy was studied world-widely and demonstrated as a novel method to treat many cancers [4]. Thus, the use of gene therapy may be a new way to treat CCRCC.

In the field of cancer gene therapy, it is well known that the success of therapy is greatly dependent on the gene delivery vectors which ensure the gene to reach target cells. Recent years, cationic polymers, which can interact with negatively charged DNA through electrostatic interaction to form nanocomplexes, are widely attempted to use as gene delivery systems. The advantages associated with this kind of vectors include that they can protect DNA from nuclease digestion, and thus enhance the gene expression within target cells, they hold low immunogenic response and can also be modified selectively, and so on [5-7]. Therefore, various cationic polymers, such as poly(L-lysine) [8] and polyethyleneimine (PEI) [9], have been synthesized and used as a gene delivery vehicle.

Among the total non-viral gene vectors, PEI, with high transfection efficiency, has bright prospects in application [9,10]. However, PEI is not fit for keeping on gene expression [10,11] due to its serious cytotoxicity. Actually, the transfection efficiency and cytotoxicity are almost antagonistic. PEI with a low molecular weight (MW), including 800-Da, 2000-Da MW or less, displays a low cytotoxicity and transfection efficiency. On the contrary, PEI with a high 25-kD MW shows higher transfection efficiency and cytotoxicity [12,13]. In order to balance the transfection efficiency and toxicity, investigators attempt to make some modifications against PEI properties. Up to now, PEI has been modified with chloroquine, polyethylene glycol (PEG), folic acid (FA), heparin and so on [4]. Furthermore, several PEI-based delivery vehicles have been used to carry DNA for gene therapy [9,14,15]. For example, a liner PEI-cholesterol conjugation was encapsulated with interleukin-12 to treat RCC mice intravenously, which was demonstrated to be effective for treatment of RCC-induced pulmonary metastases [15]. In addition, folate-target gene therapy vectors have been found to promote much higher levels of tumor-specific gene expression than nontargeted vectors [16].

In this study, we compared cytotoxicity and the transfection efficiency of the three PEI-derived materials, including poly(ε-caprolactone)-pluronic-poly(ε-caprolactone)-grafted-PEI (PCFC-g-PEI), FA-PCFC-isophorone diidocyanate-PEI (FA-PEAs) and heparin-PEI (HPEI) *in vitro *and in gene therapy carrying with Von Hippel-Lindau (VHL) on mice RCC model. These new attempts provide potential methods to treat CCRCC by VHL gene therapy, which may have a bright prospect in future.

## Methods

### Cell Lines

The human CCRCC cell line OS-RC-2 and mouse macrophage cell line Ana-1 were purchased from the Institute of Cell Biology, Chinese Academy of Sciences (Shanghai, China). Human umbilical vein endothelial cell line (HUVEC) was ordered from ATCC. All of them were maintained in RPMI-1640 media supplemented with 10% fetal bovine serum which contained 100 units/ml of penicillin and 100 units/ml of streptomycin. All cells were routinely maintained at 37°C in humidified air containing 5% CO_2_.

### Reagents

Dimethyl sulfoxide (DMSO) and 3-(4, 5-dimethylthiazol-2-yl)-2, 5-diphenyltetrazolium bromide (MTT) were ordered from Sigma Company, USA. The PCFC-g-PEI, FA-PEAs and HPEI, all were synthesized. PCFC-g-PEI was obtained by Michael addition reaction with glycidyl methacrylate-PCFC-glycidyl methacrylate (GMA-PCFC-GMA) and the 25-kD PEI [4]. PCFC was synthesized by ring-opening polymerization of ε-caprolactone initiated by pluronic 105 (poly (ethylene glycol)-poly (propylene glycol)-poly (ethylene glycol), PEG-PPG-PEG, MW = 1900 ) [17]. The cationic HPEI nanogel was conjugated by 2-kD PEI and heparin. Heparin is a biodegradable negative polysaccharide with many carboxylic groups, and PEI is a cationic polymer with many primary amine groups in its molecular structure. Thus, in presence of EDC/NHS, the reaction between heparin and PEI occurs [18]. PEAs (PCFC-IPDI-PEI) were synthesized by 2-kD PEI and PCFC copolymers using isophorone diidocyanate (IPDI) as a cross-linker [19]. The folic acid-coupled PEAs were prepared by the reaction of the activated folate ester with the amine group on the PEAs.

### Evaluation of cytotoxicity of three PEI-derived materials

The cytotoxicity of the three modified PEI-derived materials were determined by MTT assay. According to the reported methods [20], the renal cancer cell line, phagocytic cells and endothelial cells were used to detect the toxicity of three modified PEI-derived materials *in vitro*.

The OS-RC-2 cells were seeded in 96-well plates at a density of 5 × 10^3 ^cells/well in 0.1 ml growth medium and incubated overnight, then a series of concentrations of each PEI-derived material (FA-PEAs, PCFC-g-PEI and HPEI), solved in 0.1 ml fresh RPMI-1640 medium, were respectively added into each well to incubate for another 24 h. Untreated cells were used as a control. Then, 20 μl of 5 mg/ml MTT solution was added to each well for incubation 4 h. Finally, the MTT was removed, and 200 μl DMSO was added to dissolve the MTT-formazan crystals. The absorbance was measured at 490 nm by an ELISA microplate reader (Bio-Rad). Besides that, the toxicity on Ana-1 and HUVEC cells were evaluated with the same method. The cell viability (%) was calculated according to the following formula. All data were presented as the mean ± SD (Standard Deviation).

### VHL- expressing plasmid

The VHL gene was cloned into the mammalian expression vector pVITRO2-neo-mcs (Invitrogen, San Diego, CA), which can be stably transfected in mammalian cells so that the genes of interest are expressed at high levels. Moreover, it can also allow the ubiquitous and constitutive co-expression of two genes of interest. Therefore, it has usually been used as an expression vector in gene therapy [21]. The recombinant plasmid pVITRO2-VHL was validated by DNA sequencing.

### Transfection in vitro

In order to evaluate the transfection efficiency of the three PEI derivatives *in vitro*, 2 μg of GFP (green fluorescent protein) plasmids, was respectively encapsulated with FA-PEAs, PCFC-g-PEI and HPEI at different ratios to transfect into OS-RC-2 cells to detect GFP expression profiling. For FA-PEAs:GFP complexes, the weight ratio of FA-PEAs *versu*s GFP plasmids (pGFP) was 10:1, 20:1, 30:1 and 40:1 to optimize the transfection activity. Similarly, the HPEI: GFP complex, with a gradient weight ratio of 10:1, 15:1, 20:1 and 25:1, was utilized to transfect OS-RC-2 cells. As a control, based on our previous studies, 2-kD PEI was used to transfer GFP into OS-RC-2 cells at 5:1 weight ratio of PEI (2-kD) versus pGFP [18].

Furthermore, the transfection effects between PCFC-g-PEI and 25-kD PEI were also compared, and the N/P ratio of PCFC-g-PEI versus GFP was used from 5:1, 7:1, to 10:1. Correspondingly, the weight ratio between PCFC-g-PEI and GFP plasmids was 0.7:1, 1:1 and 1.3:1. This relation was acquired by the formula: N/P ratio = 7.53 × weight ratio of PEI/DNA [10]. Where N is the number of polymer nitrogen atoms and P is the number of DNA phosphorus atoms. As controls, same quantity (1:1) of 25-kD PEI and GFP was used to detect transfection activity based on our previous studies [18].

OS-RC-2 cells (1 × 10^5 ^cells/well) were seeded on 6-well plates to detect transfection activity. Before transfection, the medium 1640 in each well was replaced with 0.8 ml of fresh serum-free medium, then added a different ratio of PEI: pGFP complex to mix in 0.2 ml serum-free medium for incubation 4 h. Then the complete medium 1640 was added, and the plate was maintained at 37°C for 24 h to observe green fluorescence expression under Fluorescence Inverted Microscope (IX71, OLYMPUS).

The transfection efficiency was determined based on cell percent with GFP expressing. The number of GFP-expressing cells versus the total cell quantity in the microscope was defined as the transfection efficiency. Cell counting was performed randomly in microscopic observation scope under 10 × magnification with 3 repeats. All data were presented as the mean ± standard deviation (SD).

### Measurement of particle size and zeta potential of FA-PEAs:DNA complexes

The particle size and zeta potential of the free PEI derivatives and FA-PEAs:pVHL complexes were measured by Malvern Zetasizer 3000HS (Malvern, UK) at 25°C. Different concentration, including 1, 2, 5, 10 and 15 mg/ml of PCFC-g-PEI or FA-PEAs was separately resolved in water to test. The FA-PEAs:pVHL complexes, ranging from 5:1 to 30:1 weight ratios, were prepared by adding FA-PEAs to suitable volume of VHL plasmids to incubate at room temperature for 30 min. Then the complexes were diluted by phosphate buffered saline (PBS) buffer to 1 mL for measurement. Meanwhile, the particle size and zeta potential of PEI (2-kD):pVHL complexes, at a series of ratios from 5 to 30, were measured with the same protocol. All results were measured three times.

### RCC model and VHL-gene therapy mediated by PEI system

The following animal experiments were in compliance with all regulatory guidelines and were approved by the Institutional Animal Care and Use Committee of Sichuan University. Six to eight week-old female BALB/c nude mice were purchased from the West China Experimental Animal Center of Sichuan University (Sichuan, China). Mice were permitted one week to acclimate to their environment before studies. The CCRCC model was established in BALB/c nude mice, inoculated with 5 × 10^6 ^OS-RC-2 cells/each mouse in the right flank. Primary tumors usually became palpable on the inoculation day 9-10 and with an average 3-mm size.

On the inoculation day 11, the tumor-bearing mice were randomly assigned into 3 groups, including VHL-treated, pVITRO2 and PBS group, and each group contained 6 mice. The nanomaterial:DNA polyplexes were composed of 100 μg FA-PEAs or 100 μg HPEI solved in 0.1 ml PBS and 5 μg pVITRO2-VHL plasmids, and each mouse in the VHL-treated group was injected polyplexes by tail vein for 10 times at 2-days intervals. The mice in the pVITRO2 and PBS groups were separately injected 0.1 ml solution containing 5 μg pVITRO2:100 μg FA-PEAs (or 5 μg pVITRO2:100 μg HPEI), and 0.1 ml PBS. While, different from the FA-PEA and HPEI system, 5 μg PCFC-g-PEI:5 μg plasmids were used to transfer gene into a mouse based on the optimal transfection activity *in vitro*.

Tumor size was measured with calipers before every treatment, and tumor volumes were calculated according to the formula: width^2 ^× length × 0.52. After treatment for 10 times, all mice were sacrificed and tumor tissues were collected. One part of tissues was stored -20°C, and the other tissues were fixed in 4% formaldehyde solution for immunohistochemistry staining.

### Semiquantitative RT-PCR

Total RNA from tumor tissues was isolated using Trizol reagent (Invitrogen) to take as templates to amplify each target cDNA fragment, which was synthesized by using the cDNA Synthesis Kit (#K1622, Fermentas, USA ). The primers of VHL and β-actin for RT-PCR were designed as following. The forward primer for VHL was 5'- TCA CCT TTG GCT CTT CAG AGA TGC A -3' (25bp), and the reverse primer was 5'- GTC TTT CTG CAC ATT TGG GTG GTC T -3' (25bp). The amplified VHL fragment was 250bp in length. The designed primers for β-actin were 5'-CGG GAA ATC GTG CGT GAC-3'(18 bp, forward) and 5'-TGG AAG GTG GAC AGC GAG G-3' (19bp, reverse), and the length of the amplified cDNA was 434 bp.

PCR was performed as follows: first cycle at 95°C for 2 min, and then 30 cycles at 94°C for 45 s, 54°C for 1 min, 72°C for 1 min and a final extension cycle of 72°C for 5 min. The house-keeping gene β-actin was taken as a loading control. HEK293T cells were used as a positive control in VHL expression.

### Immunohistochemistry

The IHC analysis was performed mainly according to our previous protocols [22,23]. Tumor tissue sections with 4 μm thickness were cut from formalin-fixed and paraffin-embedded tissues for immunohistochemistry (IHC). The endogenous peroxidase was blocked with 3% H_2_O_2_, and the antigen retrieval was carried out in citrate buffer (pH6.0). The VHL expression level in tumor tissues was detected by indirect immunohistochemical staining using the labeled streptavidin-biotin method. The anti-VHL mouse monoclonal antibodies (Abcam, ab11191) were used as the primary antibodies, and the second antibody was a biotinylated anti-mouse IgG. The antigen-antibody complex was then visualized with horseradish peroxidase-streptavidin reagents and 3, 3'-diaminobenzidine solution and counterstained with hematoxylin.

### Data statistical analysis

The SPSS program (version 15.0, SPSS Inc., USA) was used for statistical analysis. Comparisons between two groups were performed by Student's t test, and comparisons among multiple groups were performed by One-way ANOVA. The difference was considered significant if p < 0.05.

## Results

### Cytotoxicity of three PEI derivatives

**Figure 1:**
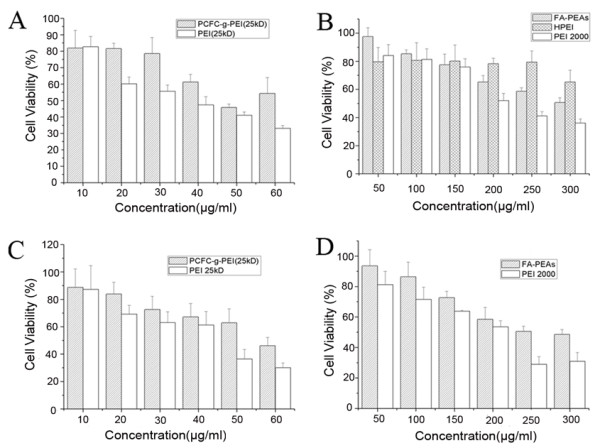
**Cell toxicity of several PEI-derived nanoparticles by MTT analysis**. The figure A-B was shown cytotoxicity on OS-RC-2 cells, and C-D indicated viability of HUVEC cells respectively treated with PCFC-g-PEI and FA-PEAs, which was respectively compared with 25-kD and 2-kD PEI. The error bars represented the standard deviation of three repeated experiments (n = 3).

The toxicity caused by the three PEI derivatives was primary evaluated in OS-RC-2 cell line. Compared with 25-kD PEI, 10, 20 and 30 μg/ml PCFC-g-PEI had no apparent cytotoxicity on cells. While the cell survival rate was approximately 50% under 30 μg/ml of 25-kD PEI incubation with cells. Therefore, the toxicity of PCFC-g-PEI was lower than its precursor, 25-kD PEI, for OS-RC-2 cells (Figure 1A), and the biocompatibility of PCFC-g-PEI was improved than 25-kD PEI.

Similarly, the toxicity of FA-PEAs and HPEI on OS-RC-2 cells was respectively compared with 2-kD PEI. As presented in Figure 1B, with the range of 50-150 μg/ml concentration, the toxicity was almost similar for three nanoparticles, FA-PEAs, HPEI and 2-kD PEI. However, the living cell number with 2-kD PEI treatment was obviously decreased when it was increased to 200-300 μg/ml concentration. Only about half of cells were alive with 200 μg/ml 2-kD PEI treatment, while 60% cells had viability even exposed to more than 200 μg/ml of FA-PEAs or HPEI. Generally, for OS-RC-2 cells, the cytotoxicity of FA-PEAs and HPEI was relatively lower when compared with its precursor, 2-kD PEI.

Generally, wide varieties of *in vitro *assays with cells should consistently reflect the possible physiologic responses to nanoparticles *in vivo*. According to the established *in vitro *assessments of nanomaterial toxicity, typically, several major cell types are used *in vitro *for testing including phagocytic, epithelial, endothelial and various cancer cell lines [20]. Therefore, besides the renal cancer cell OS-RC-2, we further observed the toxicity of the three modified PEI-derived nanoparticles on both a murine macrophage cell line, Ana-1, and a human umbilical vein endothelial cell line (HUVEC) respectively.

As a result, the toxicity of PCFC-g-PEI and FA-PEAs on Ana-1 and HUVEC cells was homoplastic with that on OS-RC-2 cells. As shown in Figure 1C, the toxicity profiling of PCFC-g-PEI on HUVEC cells was similar with that on OS-RC-2 cells. For example, with 40 μg/ml PCFC-g-PEI treatment on HUVEC, about 68% cells had viability which was mimetic to 65% on OS-RC-2 cells. Moreover, the toxicity of PCFC-g-PEI on HUVEC cells was obviously lower when compared to the precursor 25-kD PEI. Similarly, the toxicity of FA-PEAs on HUVEC cells was lower when compared to 2-kD PEI (Figure 1D). When the concentration of FA-PEAs exceeded 250 μg/ml, the percent of viable HUVEC cells was more than 50%. On the contrary, only 28.93% HUVEC was existent under 250 μg/ml of 2-kD PEI treatment. Besides that, the toxicity of PCFC-g-PEI and FA-PEAs on Ana-1 cells has homoplastic effects on OS-RC-2 cells (data not shown).

### Transfection efficiency in vitro

**Figure 2:**
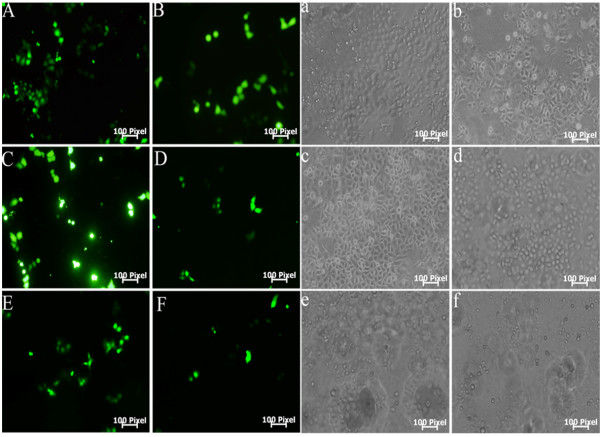
**The representative transfection image for GFP-expressing cells using PEI and PEI-derived nanoparticles as gene delivery vectors**. The GFP expression was observed under fluorescent microscope at 10 × magnification. Figure A, C, E standed for the transfection image that used PCFC-g-PEI, FA-PEAs and HPEI as a gene delivery vector respectively. And figure B, D and F represented the transfection image with 25-kD PEI and 2-kD PEI as gene vectors. Figure a-f showed the transfection image which was corresponding to Figure A-F in bright field. The scale bar represents 31.41 μm.

**Figure 3:**
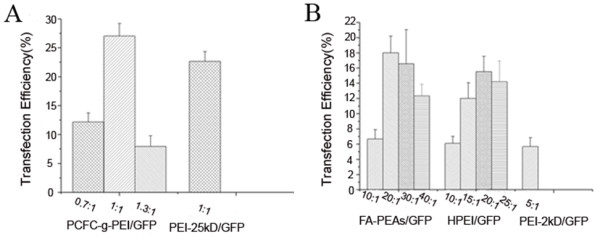
**Transfection efficiency for GFP plasmid in OS-RC-2 cells mediated by the PEI and PEI-derived nanoparticles**. The Figure 2A was shown the transfection effects of PCFC-g-PEI:GFP polyplexes at different weight ratios versus the control PEI (25 kD):GFP group. The transfection efficiency of FA-PEAs:GFP and HPEI:GFP complexes was respectively compared with the PEI (2-kD)/GFP (Figure 2B). The error bars represented the standard deviation of experimental repeats.

The GFP plasmid was used as a marker gene to detect cell transfection activity *in vitro*. The Figure 2 was the representative fluorescence profiling of positive GFP-expressing cells transfected with three PEI-derived nanoparticles. For PCFC-g-PEI, the average transfection efficiency was 12.1 ± 1.5%, 27.0 ± 2.1% and 7.9 ± 1.8% corresponding to 0.7:1, 1:1 and 1.3:1 ratio of PCFC-g-PEI:GFP complex (Figure 3A). While under same conditions, the transfection rate mediated with 25-kD PEI was 22.6 ± 1.7%, lower than 27.0 ± 2.1% which was transfected with PCFC-g-PEI:GFP at 1:1 weight ratio. Therefore, when the weight ratio of PCFC-g-PEI: GFP was 1, meanwhile, the concentration of PCFC-g-PEI was 2 μg/ml, the transfection rate of PCFC-g-PEI was highest and had no apparent toxicity on cells (<10 μg/ml). Thus, PCFC-g-PEI, with the 1:1 optimized weight ratio, had a higher transfection activity (Figure 2A-2B, Figure 3A) and a lower toxicity when compared to 25-kD PEI.

As same as PCFC-g-PEI, the transfection effect of FA-PEAs and HPEI was also related to their weight ratio too. As shown in Figure 2C and Figure 3B, the highest transfection efficacy for FA-PEAs was about 18.0 ± 2.1% at 20:1 weight ratio of FA-PEAs/GFP, while at 10:1, 30:1 and 40:1 weight ratio, there was about 6.7 ± 1.2%, 16.6 ± 4.5% and 12.3 ± 1.5% cells expressing GFP respectively. In addition, at 20:1 weight ratio, 40 μg/ml FA-PEAs showed little toxicity on OS-RC-2 cells. The similar result was also observed for HPEI, with an optimized weight ratio of 20:1, the transfection percent was 15.5 ± 2.0%, which was close to FA-PEAs (Figure 2E, Figure 3B). Differently, the cytotoxicity of HPEI was a little higher than FA-PEAs when the concentration was 40 μg/ml. However, the transfection efficacy with 2-kD PEI at 5:1 weight ratio (Figure 2D-2F, Figure 3B) was about 1/3 of HPEI or FA-PEAs at 20:1.

### Size and zeta potential of the FA-PEAs:pVHL complexes

We mainly measured the physic-chemical properties (size and charge) of each separate material, including PCFC-g-PEI and FA-PEAs, as well as the FA-PEAs:pVHL complexes. Because PCFC-g-PEI and FA-PEAs are amphiphilic polymers, both of them may form polymeric micelles through self-assembling. During the process of the micelle formation, the concentrations of polymers in solutions would be an important factor. As shown in Table 1, the size of free FA-PEAs was decreased obviously along with increase in its concentration. When the concentration was higher than 5 mg/ml, the size of FA-PEAs was stable nearby 170 nm. Meanwhile, another factor, the zeta potential was lower than 30 mV when the concentration was higher than 2 mg/ml, which indicated that the micelles may be aggregated due to concentration increased. Moreover, the size and zeta potential of free PCFC-g-PEI showed a same tendency like as the free FA-PEAs (data not shown).

**Table 1:** The particle size and zeta potential of FA-PEAs

Concentration(mg/ml)	1	2	5	10	15
Particle size (nm) (±SD)	283.9 ± 9.4	265.7 ± 7.0	172.8 ± 5.9	187.7 ± 6.2	172.0 ± 8.1

Zeta potential (mV) (±SD)	38.2 ± 1.8	21.7 ± 1.7	14.7 ± 1.8	14.6 ± 1.9	11.1 ± 0.9

SD stands for standard deviation. All results were measured three times (n = 3).

So far as FA-PEAs:pVHL complexes were concerned, as shown in Table 2, the particle size was 277.5 nm at the mass ratio (FA-PEAs versus pVHL) of 5, while the particle size was below 200 nm when the FA-PEAs/pVHL ratio exceeded 15, which indicated the plasmids were compacted with polymers more close so that the complexes were easier to enter into cells. Compared with the precursor PEI (2-kD), the size of PEI:pVHL complexes was larger than the FA-PEAs:pVHL complexes obviously at same ratio. Generally, the particle size of FA-PEAs:pVHL complexes was decreased along with the increase of the weight ratios between FA-PEAs and VHL plasmids due to the net positive electrostatic repulsion between complexes. When the optimal ratio of FA-PEAs/pVHL was 20:1, in which the transfection rate was moderate and cytotoxicity was acceptable, the complexes were kept stable. In addition, the average zeta potential of FA-PEAs:pVHL complexes at 10:1 ~ 30:1 mass ratio, was in the range of 7.1 to 31.6 mV, which was a little greater than the PEI (2-kD):pVHL complexes with 5.9 to 22.5 mV. These data indicated that the complexes became stable along with the increasing weight ratios. The strong positive surface charge of polyplex is necessary for binding to the anionic cell surface, which enables the entering of complexes into cells by cellular uptake [19].

**Table 2:** The particle size and zeta potential of FA-PEAs:VHLpolyplexes

Weight Ratio	Particle size (nm) (±SD)		Zeta potential (mV) (±SD)	
	
**(Nanoparticles *versus *VHL plasmids **^**a**^**)**	FA-PEAs: VHL	**PEI(2-kD): VHL**^**c**^	FA-PEAs: VHL	**PEI(2-kD): VHL**^**c**^
5:1 ^b^	277.5 ± 10.1	304.1 ± 5.7	4.4 ± 0.4	5.1 ± 0.3
10:1	238.8 ± 18.8	289.1 ± 8.9	7.1 ± 0.6	5.9 ± 0.3
15:1	182.4 ± 6.5	250.4 ± 4.6	11.4 ± 1.1	9.1 ± 0.2
20:1	165.4 ± 11.6	223.1 ± 12.5	15.6 ± 0.7	12.4 ± 0.9
25:1	143.5 ± 11.4	222.5 ± 9.3	22.4 ± 2.6	18.4 ± 0.5
30:1	140.3 ± 1.5	157.4 ± 5.1	31.6 ± 0.8	22.5 ± 0.7

SD stands for standard deviation. All data were measured three times (n = 3).

(a) The VHL plasmids were constant (2 μg), and the total volume of complexes was 1 ml.

(b) The concentration of free FA-PEAs was 10 μg/ml.

(c) The PEI (2-kD):VHL complexes were used as a control.

### Gene therapy effects mediated by PEI-derived nanoparticles

**Figure 4:**
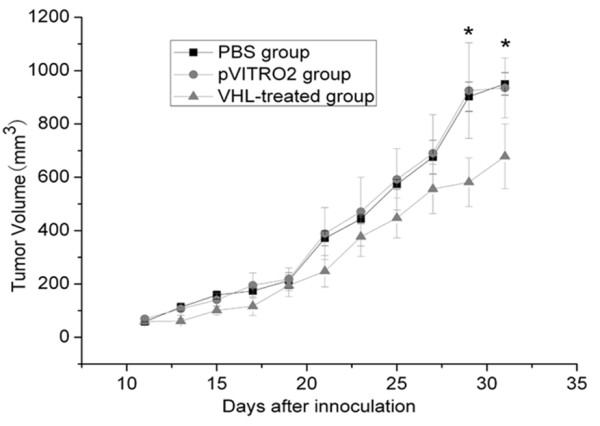
**Tumor volume of RCC-bearing nude mice treated with the FA-PEAs:VHL polyplexes**. The tumor size in each group was the mean value of six nude mice. A value of *p *less than 0.05 was accepted to be significant. *, *p *< 0.05. All the data were presented as mean ± SEM (standard error of mean), and the statistics were performed with Student's t test.

Based on the transfection effects of three modified PEI in *vitro*, we further tested whether the PEI-derived nanoparticles could efficiently transfer target gene into tumor tissues *in vivo*. The RCC tumor xenograft was established by inoculation with OS-RC-2 cells in nude mice to verify VHL gene therapy effects. Compared with the control group, the FA-PEAs:pVHL group obviously exhibited anti-tumor activity in the tumor-bearing nude mice from 21 to 31 days after implantation with OS-RC-2 cells. On day 31 post-implantation, the mean tumor volume was 678.70 ± 121.73 mm^3 ^in FA-PEAs:pVHL-treated mice, whereas tumor had 935.23 ± 112.14, 950.40 ± 41.98 mm^3 ^in FA-PEAs:pVITRO2-treated and PBS group respectively (Figure 4, p < 0.05), which indicated VHL gene therapy achieved about 30% inhibiting rate of tumor growth. Whereas there was no statistical difference between the PBS group and the FA-PEAs:pVITRO2-treated group in tumor size. These data indicated that the FA-PEAs:pVHL therapy can inhibit tumor growth in *vivo*.

Meanwhile, the mean tumor volume in HPEI:pVHL-treated mice was 881.12 ± 167.39 mm^3 ^versus 860.58 ± 124.88 mm^3 ^in PBS-treated group and 867.80 ± 91.18 mm^3 ^in HPEI:pVITRO2-treated mice. Therefore, there was no significant difference among the three groups. Similarly, when we used PCFC-g-PEI as gene delivery vectors, the inhibition effect was not obvious too (data not shown). Therefore, among the three modified PEI, the tumor was obviously suppressed when using FA-PEAs as a gene delivery vehicle.

### VHL gene expression in tumor tissues

**Figure 5:**
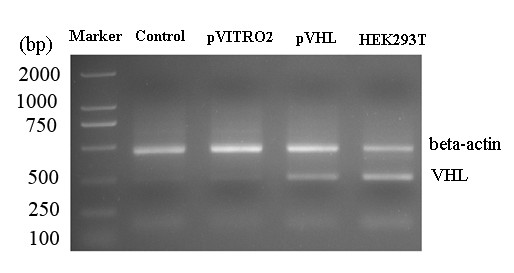
**The expression validation of VHL in tumor tissues by RT-PCR**. Marker: DNA Marker. The β-actin was taken as a loading control. The expression of VHL in 293T cells was used as positive controls.

In order to validate the expression of target gene delivered by FA-PEAs in the tumor tissues, VHL was detected by RT-PCR and immunohistochemistry. As shown in the Figure 5, VHL mRNA expression was increased obviously in VHL-treated group compared with the PBS and pVITRO2 group. Three cases of tumor tissues were performed repeatedly in each group and obtained the same results.

**Figure 6:**
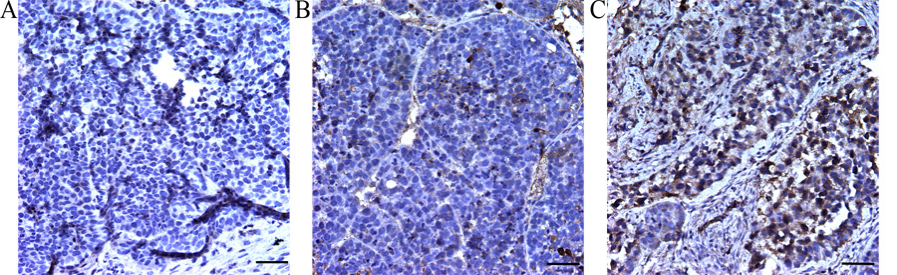
**The expression and distribution of VHL protein in RCC tissues *via *FA-PEAs mediated gene therapy**. (A)-(C) was respectively represented the PBS group, the pVITRO2 group and the VHL-treated group. The staining profiling was observed under 400 × magnification. The scale bar represents 200 μm.

Furthermore, IHC was performed to analyze the expression and distribution of VHL protein among the three groups. As shown in Figure 6, the VHL protein was abundantly expressed in cytoplasm in the VHL-treated group (Figure 6C). While under same the conditions, few or no expression activity was observed in the tumor tissues in PBS group and pVITRO2 group (Figure 6A-6B). Generally, the target gene VHL was transferred into tumor area by FA-PEAs to suppress tumor growth.

## Discussion

Recently, several PEI-modified nanomaterials, as nonviral delivery vectors, have already been extensively used to carry DNA for gene therapy. For example, the intraperitoneal injection of DNA:PEI complexes is a promising delivery method to transduce genes into disseminated cancer nodules that induced by pancreatic tumor in the peritoneal cavity [9]. Moreover, one PEI derivative, which was removed the N-acyl moieties from commercial linear 25-kD PEI, enhanced its DNA delivery efficiency 21 times *in vitro*, as well as 10,000 times in mice with a concomitant 1,500-fold enhancement in lung specificity [24]. Another water-soluble polymer, heparin-conjugated PEI exhibited significantly higher in target gene expression than 25-kD PEI [25].

Due to its intrinsic transfection properties, PEI has been used to provide the backbone of PEI-derived vector formulations. Therefore, PEI-based vector improvements are needed with regard to the efficiency and specificity of the gene transfer. In our studies, we mainly investigated the cytotoxicity and the transfection efficiency of the three PEI derivatives (PCFC-g-PEI, FA-PEAs and HPEI) *in vitro *and on a RCC model *in vivo*. The toxicity of three PEI derivatives was relatively lower than their corresponding PEI precursor on OS-RC-2 tumor cells and on other cell types, including Ana-1 and HUVEC cells. The partial reasons are probably due to the decrease in charge of complexes with decrease in primary amine amount [4]. Meanwhile, the toxicity is often associated with materials uptake by cells. HPEI has a proton-buffering effect [25] and HPEI:pVHL has higher blood compatibility and a lower cytotoxicity than PEI (25kD):pVHL.

On the other hand, with an optimized weight ratio, the transfection efficiency of PCFC-g-PEI is also a little increased than 25-kD PEI. An important part of polyplex transfection activity depends on the polyplex physico-chemical characteristics [26]. Because the PCFC complex contains a pluronic 105, in which the pluronic block copolymer could enhance polycation-mediated gene transfer *in vitro *[4,27]. Moreover, the particle size of the PEI:DNA complex was also important for its uptake by cells. For efficient endocytosis and gene transfer, the complex must be small (below 200 nm) and compact [19]. The particle size and zeta potential of the PCFC-g-PEI:DNA and HPEI:DNA complexes had been detected in our previous reports [4,18]. The size of PCFC-g-PEI:DNA complexes was relatively stable around 200 nm [4]. Therefore, when the copolymer bind to DNA, the complexes size will be condensed obviously and may be uptaken easily by cells through endocytosis. In the case of HPEI/DNA complexes, along with an increase in HPEI:DNA weight ratio, the particle size was decreased [18], which was also supported by the FA-PEAs:pVHL polyplexes.

Similarly, the transfection efficiency of FA-PEAs and HPEI was increased and the cytotoxicity was decreased compared to 2-kD PEI, especially the *in vivo *effects of VHL gene therapy mediated by FA-PEAs polymer were better than other PEI-polyplexes. Except to the same chemical structure, pluronic block copolymer, such as PCFC-g-PEI, the poly(ε-caprolactone) (PCL) segments in FA-PEAs can increase the circulating half-life [28], which is contributed to enhance gene transfer efficiency *in vitro*. Furthermore, the cross-linker IPDI stabilizes the polyplexes and prolongs circulation times at the same time [19]. In addition, the relative smaller particle size and larger zeta potential of FA-PEAs:pVHL complexes were also helpful for the nonspecific cell interaction with them. Meanwhile, FA can interact with folate receptor (FR) which is usually overexpressed in cancer cells [29]. These resulted in its better transfection efficiency *in vitro *and *in vivo*. Furthermore, both FA and heparin could be degraded easily by enzyme in cells, and the PEI amino group, which is harmful to cells, is reduced too. All these factors might contribute to low cytotoxicity of FA-PEAs and HPEI.

In order to validate the potential transport potent of the three novel modified PEI *in vivo*, we used VHL gene, a tumor suppressor gene that is usually inactivation or absence in RCC [30], to treat the RCC model on nude mice. The mean tumor volume in FA-PEAs:VHL-treated mice was decreased about 30% compared to the control group. The FR exhibits limited expression on healthy cells, but overexpression in many types of tumors, such as ovarian, colorectal and renal cell carcinomas [26,31]. Therefore, FA-PEAs:pVHL complexes could bind to FR that locates in cell surface with nanomolar affinity. The specific interactions between polyplexes and cell surface are targeted *via *a specific ligand-receptor incorporating mechanism, which is very important for *in vivo *targeting gene therapy. And the FA-PEAs:VHL complexes might be released into cytosol through endocytosis [32]. This is may be the most important reason that FA-PEAs:VHL exhibits an obvious therapeutic efficacy.

## Conclusions

The three modified PEI-derived biomaterials, including PCFC-g-PEI, FA-PEAs and HPEI, had an increased transfection efficiency *in vitro *and obviously lower toxicities compared with their precursor PEI with 25-kD or 2-kD molecule weight, and the gene therapy effects on RCC model mice were obvious by using FA-PEAs:pVHL complexes to treat tumor. Therefore, FA-PEAs may be a potential gene transfer system to carry VHL gene to treat RCC in future.

## List of abbreviations

DMSO: dimethyl sulfoxide; FA: folic acid; FA-PEAs: FA-PCFC-isophorone diidocyanate-PEI; GFP: green fluorescent protein; HPEI: heparin-PEI; IHC: immunohistochemistry; IPDI: isophorone diidocyanate; MTT: 3-(4, 5-dimethylthiazol-2-yl)-2, 5-diphenyltetrazolium bromide; PEG: polyethylene glycol; PEI: polyethyleneimine; PCFC-g-PEI: poly(ε-caprolactone)-pluronic-poly(ε-caprolactone)-grafted-PEI; RCC: renal cell carcinoma; VHL: Von Hippel-Lindau.

## Competing interests

The authors declare that they have no competing interests.

## Authors' contributions

XZ performed the experiments and wrote the paper draft; LS conceived, instructed the experiments and revised the paper; SG and XX performed experiments and analyzed data; ZX and ZP collected and validated tissue samples; WH and GQ prepared PEI-derived material; QZ guided the PEI synthesis; WY provided experimental devices and gave suggestions on this project. All authors read and approved the final manuscript.
